# Glymphatic System Dysregulation as a Key Contributor to Myalgic Encephalomyelitis/Chronic Fatigue Syndrome

**DOI:** 10.3390/ijms262311524

**Published:** 2025-11-27

**Authors:** Mohsen Nemat-Gorgani, Michael Anthony Jensen, Ronald Wayne Davis

**Affiliations:** Stanford Genome Technology Center, Department of Biochemistry, Stanford University, Palo Alto, CA 94304, USA; m.a.jensen@stanford.edu (M.A.J.); ron.davis@stanford.edu (R.W.D.)

**Keywords:** ME/CFS, neuroimmune, neurological disorders, sleep disturbance, AQP4 water channels, glymphatic system, glymphatic dysfunction

## Abstract

Defined by the World Health Organization as a neurological disorder, Myalgic Encephalomyelitis/Chronic Fatigue Syndrome (ME/CFS) is a disabling illness, affecting millions of people worldwide. First reported in the early nineteenth century, ME/CFS is uniquely characterized by a wide array of symptoms, including fatigue, brain fog, post-exertional malaise (PEM), sleep dysfunction, and orthostatic intolerance (OI). Despite decades of extensive research, there are no effective medical treatments or simple diagnostics for ME/CFS, with an estimated 90% of patients remaining undiagnosed. The recently discovered glymphatic system, a lymphatic analog of the brain, is believed to be responsible for the removal of toxic metabolic wastes accumulated in the course of daily activities, primarily during sleep. A link between glymphatic dysfunction and some neurological disorders such as Alzheimer’s disease has already been established, raising the possibility of its involvement in ME/CFS. Accordingly, we believe the ME/CFS medical/scientific community will be interested in seriously considering GD an important contributor to its pathophysiology. If so, therapeutics that modulate glymphatic function may also benefit patients with ME/CFS.

## 1. Introduction

A condition resembling Myalgic Encephalomyelitis/Chronic Fatigue Syndrome (ME/CFS) was recognized as an unknown post-viral illness following a series of outbreaks worldwide in the 1930s [1]. Since 1969, ME/CFS has been classified as a neurological disease in the International Classification of Diseases by the World Health Organization (WHO, ICD G93.3) [2], which is described as a “dysfunction of integrative brain regions” by the National Institutes of Health [3]. Our own interest in the neurological aspect of ME/CFS pathophysiology was initiated upon encountering the presence of autoantibodies in the plasma samples of some patients where demyelination may be occurring [4].

Major symptoms of ME/CFS include fatigue, sleep disturbance, post-exertional malaise (PEM), orthostatic intolerance (OI) and brain fog [5,6]. Among these, PEM and sleep disturbance are generally considered hallmark symptoms, while there are other symptoms that overlap with fibromyalgia and Long COVID, to name a few chronic conditions [7,8], with 49% of Long COVID patients meeting the ME/CFS criteria [9]. Degree of illness also ranges from mild to severe, and in many cases the patients are bedridden [10], suggesting major molecular changes leading to a complex array of symptoms [11].

A prominent feature of ME/CFS is PEM, often called a “crash” [10], which results from overexertion after performing even normal physical or mental activities, and which may be related to heightened innate immune response and neuroinflammation believed to be occurring in the patients [12]. Feeling unrefreshed after sleep is a common and defining symptom, and persistence of the “crash” phase is often long and painful [13,14]. Therefore, by understanding the root pathophysiology of ME/CFS, not only may chances of recovery be increased, but also it may be possible to avoid the “crash” altogether. While physicians can identify patients with ME/CFS using special symptom metrics (e.g., Canadian Consensus Criteria), there are currently no simple diagnostic tests [15,16,17]; furthermore, there is no cure for ME/CFS, and treatment is difficult, requiring a cocktail of medications and changes in diet and lifestyle, which cannot address all symptoms. Therefore, the biggest challenge facing the scientific community is finding a common denominator that may explain the myriad of symptoms experienced in ME/CFS. This would provide the necessary roadmap to its etiology where the puzzle pieces of ME/CFS may finally be assembled. Ultimately, this would translate into a better understanding of the disease that may lead to more effective treatment, a prospect for recovery from the “crash,” and the possibility of getting closer to a cure.

As a complex, heterogeneous, and multisystem disease, ME/CFS has been examined from a wide array of perspectives, exploring various potential contributing factors and biological abnormalities. With the recent discovery of the glymphatic system (the brain lymphatics for waste removal) [18], its dysfunction has been described to be the root cause of many neurological pathologies, including Alzheimer’s disease [19]. With the understanding that ME/CFS is a complex neurological disorder (WHO, ICD G93.3) [2], we investigated the possibility of its association with glymphatic system function. Accordingly, here we propose that a dysfunctional glymphatic system (GD) also plays an important role in most, if not all, symptoms in ME/CFS. While some connections between ME/CFS and recovered COVID-19 patients have already been described in association with GD [20,21], we attempt to provide a more comprehensive display.

## 2. The Glymphatic System

**Figure 1:**
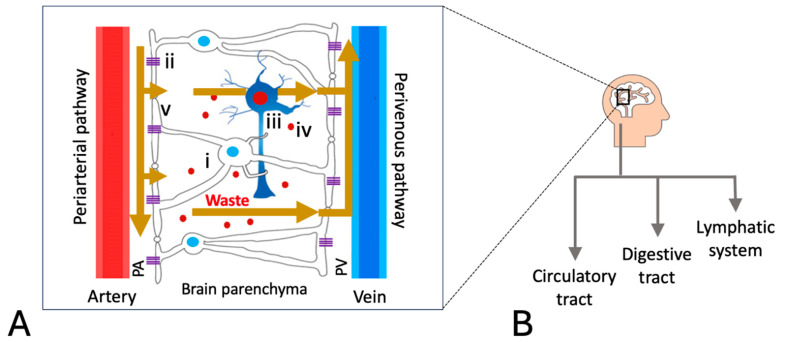
(**A**) Schematic presentation of a functional glymphatic system with clearance of protein aggregates and metabolic wastes mediated through fluid movement from CSF into the brain and from ISF out of the brain (i, astrocyte; ii, AQP4 channel; iii, neuron; iv, metabolic waste; v, astrocyte end-feet; PA, periarterial space; PV, perivenous space) and (**B**) downstream pathways interconnected with the glymphatic system [23].

The glymphatic system (glial–lymphatic) is the lymphatic analog of the brain necessary for the removal of metabolic wastes (e.g., lactate, glutamate, reactive oxygen species, ammonia, and protein aggregates such as tau and β-amyloid) [18,19]. This unique system plays a critical role in brain homeostasis by facilitating cerebrospinal fluid (CSF) and interstitial fluid (ISF) exchange through directional fluid movement via the periarterial and perivenous pathways (Figure 1); here, efflux fluids drain into the meningeal lymphatic vessels and eventually exit from the central nervous system (CNS) to the bloodstream [22].

**Figure 2:**
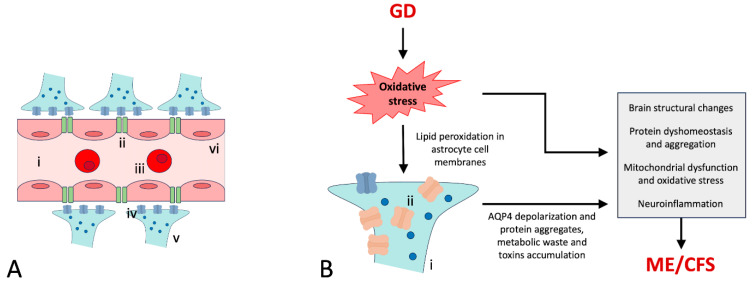
(**A**) Schematic presentation of the blood–brain barrier and AQP4 channel localization, which enable astrocytes to mediate brain fluid homeostasis under normal conditions (i, artery; ii, tight junction; iii, RBC; iv, AQP4 water channel protein (“polarized distribution”); v, astrocyte end-feet (with water molecules); vi, endothelial cells of the blood–brain barrier), and (**B**) dysfunctional astrocyte showing AQP4 depolarization due to oxidative stress (i, depolarized AQP4; ii, end-feet of astrocytes).

Also, unique to the glymphatic system are perivascular spaces containing astrocytes (glia), which are essentially housekeeping cells that maintain neuronal homeostasis. The astrocyte end-feet processes that wrap around blood vessels in the brain comprise aquaporin-4 (AQP4) water channels (Figure 1B). These channels regulate fluid balance across the blood–brain barrier (BBB) and facilitate the flow of CSF through the perivascular spaces of the brain, thereby contributing to the waste removal process (Figure 2A).

While the glymphatic and meningeal lymphatic systems work together to remove waste products from the brain, other factors are involved to assist with the cleanup process. For example, damaged or misfolded proteins can form amyloid structures (protein aggregates) that may build up and block the AQP4 channels and hinder CSF–ISF exchange. These amyloid structures are normally broken down by lysosomal proteases as part of autophagy, a cellular waste removal mechanism that works together with the glymphatic system [24]; this is believed to be impaired in ME/CFS [25]. Furthermore, degradation of the amyloid structures may also take place by catalytic antibodies, or “catabodies” [26].

## 3. Blood–Brain Barrier Dysfunction

The BBB is vital to the CNS, protecting it from pathogens and toxins that are circulating in the blood. It is a restricted structure between the brain parenchyma and circulating blood. The breakdown of the BBB has been reported to occur in many pathologies, such as neurodegenerative- [27], autoimmune- [28], and infection-related diseases [29,30,31].

Composed of astrocytes, pericytes, and brain endothelial cells, the structural anatomy of the BBB and the glymphatic system partially overlap [32] and are complementary mechanisms [33], with GD being a possible risk factor for BBB dysfunction [34]. Moreover, BBB dysfunction and neuroinflammation have been described in ME/CFS [35,36,37].

The endothelial cells are the major structural and functional components of the BBB and are sealed together with tight and adhesion junctions (Figure 2A). A number of studies have indicated that elevated levels of reactive oxygen species (ROS) can affect BBB integrity by a number of mechanisms, the main ones of which are altering expression, altering distribution, and causing modification of tight-junction proteins [38,39,40]. BBB dysfunction can cause an accumulation of toxic substances in the interstitial fluid surrounding neurons and glial cells. This in turn may lead to GD, which can then result in pathological changes (see below).

## 4. Glymphatic System Dysfunction (GD)

A number of reports have indicated that immune system dysregulation and neuroinflammation can impair the glymphatic waste removal process. Immune cells and inflammatory factors can accumulate in perivascular spaces, disrupting glymphatic function and leading to a cycle of inflammation that further damages the system and the brain. Conversely, GD can result in the reduced clearance of inflammatory mediators, exacerbating neuroinflammation [41,42,43].

There are several other factors that may contribute to a dysfunctional glymphatic system, including obesity, depression, and stroke [44,45,46], in addition to neurological disorders [19]. Alzheimer’s disease, for example, can be the result of amyloid buildup due to GD [18,19,23]. Disrupted sleep and blood flow can also significantly impede the brain’s waste clearance process; when there is waste overaccumulation, this promotes a cascade of events, from protein fragment aggregation to dysautonomia, neuroinflammation, and cognitive impairment [44,47,48].

## 5. Role of AQP4 in the Glymphatic System

AQP4 water channels are the primary pathway for water movement into the brain’s interstitial space, and when dysfunctional, this process is significantly reduced. AQP4 proteins are expressed at the plasma membrane of astrocytes throughout the central nervous system and are essential to the functioning of the glymphatic system. They facilitate the exchange of fluids between CSF and ISF (Figure 1) [49], thereby playing a crucial role in preserving fluid homeostasis of the brain. Reduced function of AQP4 [50] under pathological conditions substantially inhibits proper waste clearance from the brain, leading to a buildup of amyloid-β, tau, and toxic metabolites such as lactate, causing GD and neurodegenerative diseases such as Alzheimer’s and Parkinson’s, as well as brain swelling from injuries [51]. Anti-AQP4 autoantibodies may also target AQP4 components, as observed in the CNS autoimmune disease neuromyelitis optica (NMO) [52]. This attack may lead to inflammation of and damage to the optic nerves and spinal cord. Furthermore, the autoantibody is used as a key diagnostic marker for the disease. We also hypothesize that anti-AQP4 autoantibodies may be involved in the pathophysiology of ME/CFS as a neuroimmune disorder.

One of the most consequential effects of ROS accumulation, under impaired glymphatic function [53], is astrocyte membrane damage by lipid peroxidation, due to its high polyunsaturated fatty acid content [54]. This can affect AQP4 polarization, with AQP4 being highly concentrated in the end-feet of astrocytes (Figure 2). The arrangement occurring in a normal, healthy situation is crucial for the glymphatic system since it enables the rapid and efficient flow of CSF and ISF to clear waste from the brain. Accordingly, astrocyte depolarization may initiate a vicious cycle, resulting in more severe GD and leading to the accumulation of toxic byproducts and a higher risk of neurodegenerative diseases.

A current hypothesis in ME/CFS research suggests that because of adrenergic dysfunction, chronic high levels of norepinephrine may interfere with AQP4 regulation, leading to potential impairment of glymphatic function [55]. Because of its central role in waste clearance, AQP4 is a target of interest for developing therapies to treat a range of neurological and psychiatric disorders [56,57,58].

## 6. GD in the Pathophysiology of ME/CFS

ME/CFS is a poorly understood disease, affecting ~1% of the world population, according to a recent report [59], and more than 9 in 10 people may not be diagnosed by a doctor [60]. It is a debilitating, multisystem chronic illness characterized by disturbances in the CNS, cell energy metabolism, and the immune and cardiovascular systems [61]. It is also associated with chronic inflammation, gastrointestinal dysfunction, and cognitive impairment [62]. In addition to these conditions, reduced cerebral blood flow [63,64] and a dysfunctional glymphatic system [65,66,67,68,69], could potentially explain many symptoms of ME/CFS.

Next, we attempt to explore some of the key symptoms and show how they may be traced back to specific dysfunctions of the glymphatic system.

## 7. Infection

Our current understanding is that in the majority of ME/CFS cases, the onset is triggered by an infection [70,71], which can also cause GD [21,72]. Impairment of the immune system, the production of autoantibodies, inflammation, mitochondrial dysfunction, and damage to the nervous system are some of the key elements of an infection that may contribute to ME/CFS pathogenesis [62,73]. BBB dysfunction and neuroinflammation, which are connected to GD, could occur concomitantly as a result [31,74,75]; moreover, infection can cause astrocyte instability at the BBB and promote displacement of the AQP4 channels [76].

Herpesviruses have been frequently associated with ME/CFS, and of these, infections with Epstein–Barr virus (EBV), human herpesvirus-6 (HHV-6), and cytomegalovirus (CMV) are considered some of the triggering factors for ME/CFS [77]. After an acute infection, these viruses may persist for life in the body and may later be reactivated.

Epigenetic silencing is also key to preventing viral reactivation [78], with both lytic and non-lytic cycles being controlled by the host’s enzymatic, metabolic, and other regulatory factors. Of the many different types of viruses involved in ME/CFS, reactivation mechanisms of EBV have been of particular interest. Accordingly, a large number of factors, including metabolites [79], stressors [80], and several biological agents, such as bacteria, viruses, and fungi, have been reported to cause reactivation [81]; each may be connected to GD, especially in the context of neurological symptoms [43].

Neuroinflammation of the brain caused by GD releases proinflammatory cytokines (e.g., TNF-α, IL-1β, and IL-6) that can enter circulation, reach other parts of the body, and trigger systemic inflammation/immune dysregulation [43]. As part of a vicious cycle, tissue damage at these remote locations then releases more proinflammatory cytokines that circulate back to the brain and perpetuate GD. Furthermore, polyautoimmune disorders (e.g., Sjögren’s syndrome and systemic lupus erythematosus) exacerbate the process. Comorbidities are also a major factor, especially with multi-symptom diseases like ME/CFS, and though considered primarily a neurological disease, ME/CFS does have an autoimmune component that feeds into the cycle and may contribute to systemic immune dysregulation [62].

## 8. Platelet Hyperactivation

Platelets play a crucial role in forming blood clots to stop bleeding after vascular injury. Various studies in ME/CFS have indicated platelet hyperactivation and defects in clotting by fibrous amyloids [82]; accordingly, fibrin amyloid microclots are resistant to breakdown and could limit oxygen delivery to tissues by obstructing blood flow. Also, cerebral blood flow issues have been reported in ME/CFS [83], which can cause GD [84], thereby linking the two pathophysiologies.

## 9. Neuroinflammation

Neuroinflammation has been described in both ME/CFS [85] and GD [86]. Here, oxidative stress, a major symptom in ME/CFS (described above), can cause GD in a number of ways, including astrocyte dysfunction [87] and NLRP3 inflammasome activation [86]. Moreover, accumulation of protein aggregates, such as amyloid-β, an important outcome of GD, may cause the release of pro-inflammatory cytokines [88] and result in neuroinflammation [89]. Of these cytokines, IFN-α-induced persistent fatigue is believed to be associated with ME/CFS [90].

## 10. Heavy Metal Toxicity

Heavy metal toxicity can affect multiple organs and has been described as being related to both GD [91] and ME/CFS [92]. Toxicity by heavy metals, such as cadmium, lead, and mercury, can cause overproduction of ROS, oxidative stress, lipid peroxidation, inflammation, antioxidant depletion (e.g., of glutathione), disruption of astrocyte function, and protein inactivation and aggregation [93]. There are a number of reports, too, indicating that heavy metal toxicity may contribute to more common symptoms of ME/CFS, most notably sleep disturbance [92].

## 11. Oxidative Stress, Hypoxia, and Endothelial Dysfunction

**Figure 3:**
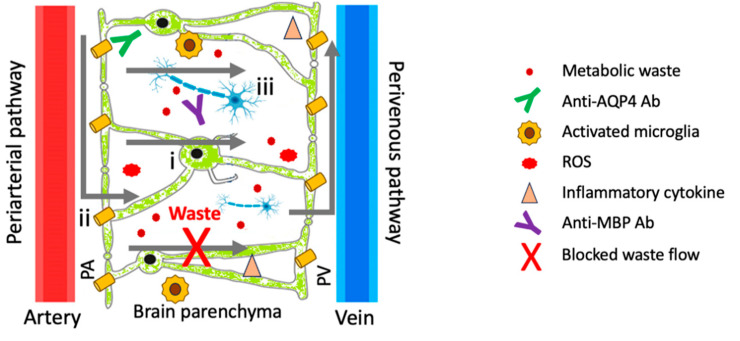
Schematic representation of GD/chronic neuroinflammation. Here, metabolic waste accumulation upon GD can promote the generation of ROS and proinflammatory cytokines, thereby activating NLRP3 inflammasome in microglia, which leads to neuroinflammation and neurodegeneration. i, Astrocyte; ii, AQP4 channel; iii, neuron.

Oxidative stress is believed to be a significant contributor to the pathophysiology of ME/CFS [94,95]. Because the brain consumes ~20% of the body’s total oxygen, it is extremely sensitive to oxidative damage due to its high cell membrane polyunsaturated fatty acid content [54] and weak antioxidant defense [96]. A dysfunctional glymphatic system therefore may result in an accumulation of ROS in the brain exceeding the capacity of the antioxidant system to neutralize harmful free radicals, the consequences of which include lactic acidosis and increased production of proinflammatory cytokines that lead to both BBB dysfunction [97] and neuroinflammation/GD (Figure 3) [42,98].

For proper CSF–ISF exchange, AQP4 must be localized to the end-feet processes in direct contact with the blood vessels. This “polarized distribution” of AQP4 (Figure 2A iv) [99] facilitates CSF circulation and efficient metabolic waste removal. Any disruption of AQP4 expression or its displacement can interrupt the CSF–ISF exchange [76], causing an accumulation of waste products. Accordingly, a loss of AQP4 polarization has been shown to trigger a wide range of brain pathologies, including stroke [100] and Alzheimer’s disease [57]. Hypoxia can be a direct cause of oxidative stress due to increasing the production of ROS, which is primarily due to mitochondrial dysfunction [101,102].

**Figure 4:**
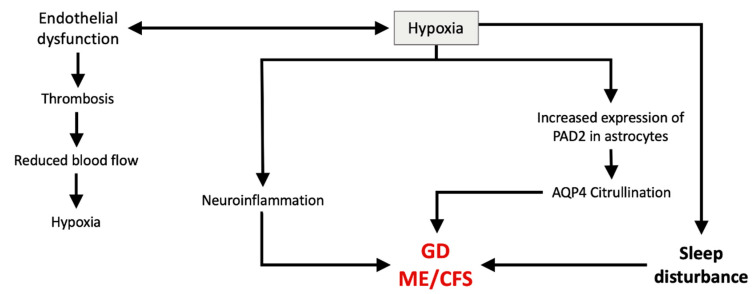
The bidirectional relationship between hypoxia and endothelial dysfunction, which may play an important role in the pathophysiology of ME/CFS and GD.

There is a bidirectional relationship between hypoxia and endothelial dysfunction, where endothelial dysfunction can be driven by hypoxia [103] and vice versa [104] (Figure 4). Cerebral blood flow abnormalities have been reported in ME/CFS [83], resulting in hypoxia due to an inadequate supply of oxygen; this impairs the glymphatic system [105] and leads to neurological symptoms in ME/CFS such as cognitive difficulties and brain fog [61]. Furthermore, neuroinflammation [106] and sleep disturbance [107] can be the result of hypoxia.

Hypoxia may also have a significant role in the autoimmune process. In response to low oxygen levels, hypoxia-inducible factor 1a (HIF-1a) regulates gene expression of the transcription factor NF-kB and peptidyl arginine deiminase (PAD) [108].

PAD is involved in citrullination of protein structures by converting arginine residues to citrulline, an unusual amino acid. The resulting post-translational modification (PTM) can change the structure and conformation of the tissue substrate and thus trigger an immune response. As an example, autoantibodies raised against citrullinated myelin basic protein could cause demyelination [4]. As related to AQP4, PAD2 expression has been observed in astrocytes [109] and was found to be increased under different environmental conditions, including hypoxia [110]. This would suggest that autoantibodies (anti-AQP4) may be produced against some of the AQP4-modified components, affecting its function and causing possible impairment of the glymphatic waste clearance mechanism.

## 12. Mitochondrial Dysfunction and Fragmentation

**Figure 5:**
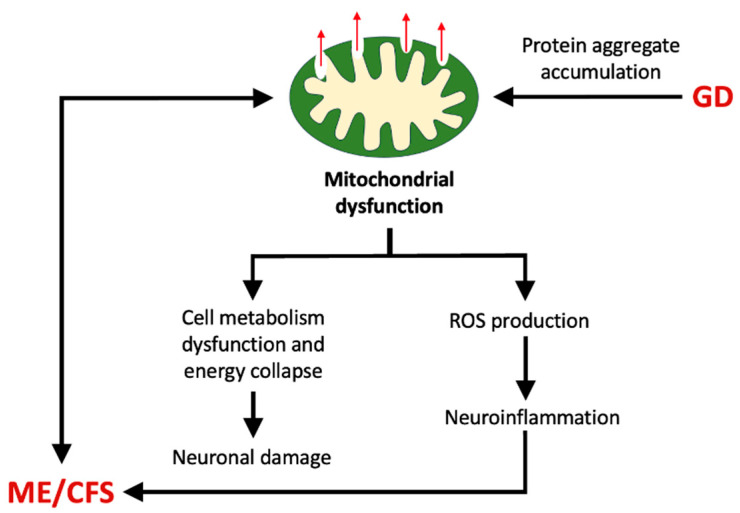
Proposed relationship of mitochondrial dysfunction between GD and ME/CFS. An accumulation of protein aggregates induced by GD may cause mitochondrial membrane permeabilization and energy collapse, which could promote oxidative stress and neurodegeneration.

Mitochondrial dysfunction is believed to play an important role in the pathophysiology of ME/CFS [111]. When the glymphatic system is impaired, it may result in protein (e.g., amyloid-β) aggregate accumulation, which further facilitates mitochondrial membrane permeabilization and the loss of mitochondrial function (Figure 5) [112,113,114,115,116].

Mitochondrial dysfunction can also increase the level of ROS and thereby cause neuroinflammation [117]; this has been reported in both ME/CFS [118] and GD [86] (Figure 5). A common feature of mitochondrial dysfunction is fragmentation, which often results from an imbalance between the processes of fission and fusion [119]. Both oxidative stress and viral infection (e.g., HHV-6) are believed to be responsible for mitochondrial fragmentation in ME/CFS [120,121]. Although mitochondrial fragmentation has not been specifically described as being related to the glymphatic system, it may occur as part of the general mechanism of mitochondrial dysfunction reported for GD [47].

## 13. Post-Exertional Malaise (PEM)

As a hallmark symptom that is most significant to ME/CFS, PEM is characterized by a disproportionate worsening of fatigue, pain, and cognitive dysfunction, and is a manifestation of a “crash.” A “crash” may last for days, weeks, or longer, even after minor exertion. Endothelial dysfunction [122], impaired oxygen delivery issues [83,123], disruption in metabolic pathways with the accumulation of waste products [124], and problems with red blood cell (RBC) biomechanics may be involved [125,126,127], which may also affect glymphatic function. Of these, abnormal cerebral blood flow [83] and an accumulation of metabolic wastes such as lactate can hamper the glymphatic clearance system. Also, endothelial dysfunction can impact the glymphatic system by impairing the BBB [41].

Recent studies of patients with Long COVID, particularly those experiencing PEM, have found amyloid-containing deposits in skeletal muscle tissue [14]. These can have major consequences related to the glymphatic system since amyloid structures from peripheral circulation can pass through a dysfunctional BBB, a process that is believed to be bidirectional [128], in addition to having the capacity to cause its disruption [129]. Interestingly, fibrin amyloid microclots have been observed in ME/CFS patients [82], similarly to Long COVID, as described above. Based on these observations, we propose that GD and the pathophysiology of PEM in ME/CFS are connected.

## 14. Lactic Acidosis

Lactate is normally cleared from the brain via the glymphatic pathway as part of its metabolic waste removal mechanism (Figure 1) [66,98]. Accordingly, lactate accumulation in the brain may occur under GD, contributing to acidosis. Lactate is also believed to be elevated in ME/CFS, even in resting conditions, and is correlated with the severity of PEM [124]. Increased brain lactate levels may also occur as the result of low blood flow to the brain [130] or by impaired pyruvate dehydrogenase activity in ME/CFS [131]. Moreover, lactic acidosis is a marker of mitochondrial dysfunction [132,133], occurring when mitochondria are unable to perform oxidative phosphorylation effectively. Impaired mitochondrial energy metabolism has been reported for ME/CFS [134], which was further confirmed by earlier studies of elevated brain lactate [135].

## 15. Brain Fog

Brain fog is a primary symptom of ME/CFS, which many patients find profoundly debilitating. Described as a feeling of “mental fogginess” and as difficulty thinking, focusing, and remembering [5], it is a subjective feeling of cognitive impairment and is generally believed to be a symptom of neurological dysfunction [136]. A connection to BBB dysfunction has recently been found in Long COVID [137,138,139]. Brain fog has also been described in relation to GD and COVID-19 [140,141]. A number of factors believed to cause brain fog in ME/CFS, including oxidative stress [142], neuroinflammation [35], BBB dysfunction [139], reduced cerebral blood flow [136], and lactic acidosis [143], have also been described to induce glymphatic dysfunction, as discussed above.

## 16. Dysautonomia and Vagus Nerve Stimulation

**Figure 6:**
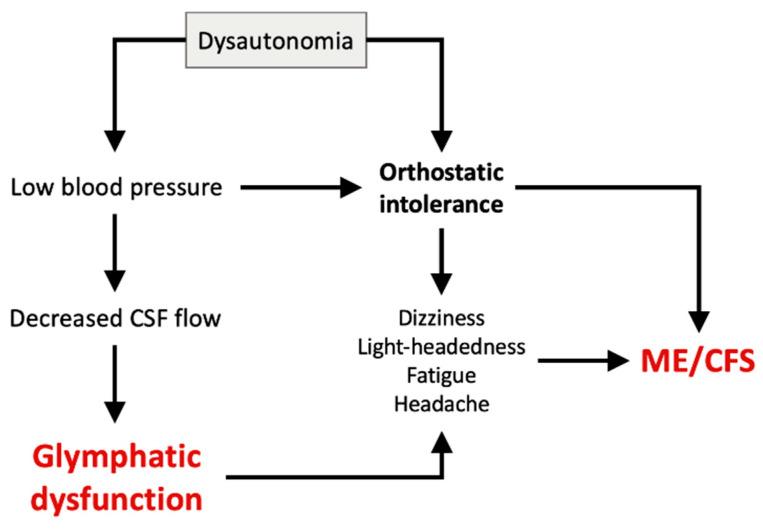
Proposed relationship between dysautonomia, GD, and ME/CFS. Dysautonomia may cause orthostatic intolerance, leading to ME/CFS. A lowering of blood pressure can cause a decrease in cerebral blood flow and thereby impact the glymphatic system.

The autonomic nervous system (ANS) regulates functions of the internal organs (e.g., cardiac output/heartrate, blood pressure, temperature, and digestion) by maintaining a balance between the sympathetic and parasympathetic nervous systems. Dysautonomia is a core feature of ME/CFS [144,145], potentially contributing to a number of key symptoms, including Postural Orthostatic Tachycardia Syndrome (POTS) and orthostatic hypotension, as part of orthostatic intolerance (OI) [146,147] (Figure 6). Symptoms of dysautonomia, which include dizziness, lightheadedness, fatigue, and headache, limit daily activities for many people with ME/CFS; these symptoms are known to worsen upon standing and are relieved by lying down. Reduced heart rate variability (HRV) [148], which has significance in relation to fatigue severity in ME/CFS [149], are included in this category of symptoms. The ANS also plays a crucial role in regulating the glymphatic system where dysautonomia may promote glymphatic failure [150,151,152].

**Figure 7:**
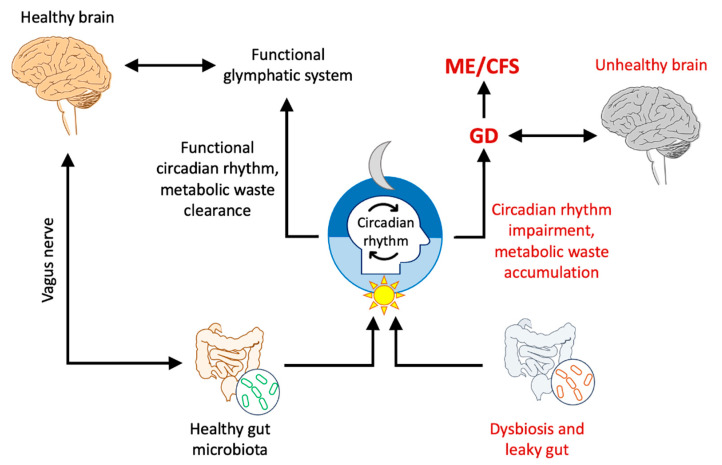
A potential role of the gut microbiota in the glymphatic system, mediated by circadian rhythm regulation. An impairment in the circadian rhythm, mediated by dysbiosis, can significantly affect glymphatic clearance function and lead to neurological disorders, including ME/CFS.

As a major part of the ANS, the vagus nerve influences functions such as heartrate, breathing, and digestion (Figure 7). Reports indicate that vagus nerve dysfunction may be part of the pathophysiology of ME/CFS; here, some patients may benefit from vagus nerve stimulation by alleviating symptoms such as fatigue and PEM [153].

The glymphatic system is also strongly associated with the vagus nerve, and its stimulation is believed to accelerate the CSF flow into the brain parenchyma and enhance the CSF–ISF exchange [150]. The vagus nerve also plays a crucial role in the brain–gut axis, a bidirectional communication between the brain and the digestive system [154] (Figure 7); its stimulation may help restore balance to the gut microbiome [153], which is important to the pathophysiology of ME/CFS and the glymphatic system.

## 17. Circadian Rhythm and Sleep Dysfunction

Unrefreshing sleep is a key hallmark of ME/CFS, affecting up to 95% of patients [155]. During sleep, the glymphatic system clears CNS metabolite wastes and other harmful substances that accumulate throughout daily brain activity [156], with astrocytes playing a major role [157]. The glymphatic system is primarily active during non-rapid eye movement (NREM) sleep [158] at a point when ME/CFS patients have greater sleep instability [159]. Moreover, the glymphatic system is influenced by the circadian rhythm, and a malfunctioning of its activity may hinder efficient removal of toxic proteins; this promotes aggregation, with toxicity to neurons, resulting in neurological disorder (Figure 7) [160]. Accordingly, the glymphatic system and sleep dysfunction appear to be closely linked, where the CSF–ISF exchange is compromised by sleep cycle abnormalities [161]. Oxidative stress and neuroinflammation, two of the consequences of GD [42,53], occur in ME/CFS [94,95] and can also impact quality of sleep [162,163].

## 18. Dehydration, Hypovolemia, and Cardiac Output

Dehydration can be a serious problem in some patients with very severe ME/CFS as a consequence of impaired swallowing [164]. These patients are in a debilitated state and unable to eat or drink, leading to malnutrition and dehydration. Dehydration can be a significant problem since it may lead to hypovolemia, affecting cardiac output as a result [165]. As a consequence, individuals with ME/CFS may exhibit lowered cardiac output due to hypovolemia [166]. Also, since the glymphatic system requires adequate fluid volume for efficient waste clearance, hypovolemia can potentially disrupt proper CSF–ISF exchange, leading to GD in these patients [167].

## 19. Gut–Brain Axis Dysregulation and Dysbiosis

The gut–brain axis is a bidirectional communication system between the gastrointestinal tract and the CNS. A healthy gut microbiome (balanced community of microorganisms, including bacteria, viruses, and fungi) is believed to be essential for normal physiological activities, including the circadian rhythm and sleep (Figure 7) [168].

A disruption in gut–brain communication, which has been reported for ME/CFS [169], has also shown to affect the glymphatic clearance system [168]. Moreover, dysbiosis, an imbalance in the composition of the gut microbiota, has been reported for both ME/CFS [170] and glymphatic dysfunction [171], causing neurological problems [172].

## 20. Idiopathic Intracranial Hypertension (IIH)

IIH, a condition where there is elevated pressure within the skull that causes fatigue and headaches, has been reported in ME/CFS [173]. This corresponds to increased intracranial pressure (ICP), which is affected by high CSF buildup in these patients [174], some of whom may benefit from IIH treatments, including surgery [175]. Craniocervical instability, which involves a weakening of the ligaments supporting the head and upper neck, and Ehlers–Danlos syndrome, both frequently reported in ME/CFS [174,176], are believed to be contributing factors to IIH [177]. IIH is also associated with impaired glymphatic function [178,179].

## 21. Brain Temperature

Since the brain is naturally hotter than the rest of the body, blood circulation acts to “cool” it by removing “hot blood” [180,181]. Abnormal cerebral blood flow and neuroinflammation are believed to be two of the main contributing factors in causing elevated brain temperature in ME/CFS [182]. Hyperthermia may also hamper glymphatic function by obstructing the flow of CSF through perivascular spaces (Figure 3), thereby reducing the efficiency of waste removal in the brain [183].

## 22. Brain pH

Brain pH regulation is crucial for neuronal function. To maintain acid–base balance, the brain has developed various mechanisms, including a buffering system in astrocytes [184]. Some studies suggest a potential decrease in the brain pH of ME/CFS patients, possibly due to a buildup of lactic acid [130]; this is also related to GD, since the glymphatic system plays a key role in cleaning waste products (e.g., lactate) from the brain [66,98] (see “Lactic Acidosis,” above).

## 23. GD Modulation for ME/CFS

Due to potential GD in ME/CFS, patients may benefit from treatments that improve the CSF influx/CSF–ISF efflux process in the brain to remove waste. For example, modulation of the glymphatic system can be accomplished by targeting perivascular CSF flow and AQP4 water channels, and by monitoring tracer flow in the brain parenchyma [32,185]. While certain lifestyle and behavioral modifications (e.g., improved sleep, exercise, meditation [156,186,187]) have been suggested, there are noninvasive techniques that have been tested with success in both GD and ME/CFS, including electroacupuncture [188,189] and repetitive transcranial magnetic stimulation (rTMS), which is used to facilitate cortical neural activity in the glymphatic system [190,191,192]. Moreover, therapeutics (noradrenaline and specific serotonergic antidepressants) for GD and ME/CFS have been associated with increased astrocytes and upregulated AQP4 expression; these include ketamine [187,193] and mirtazapine (improves glial cell line-derived neurotrophic factor production) [65,186]. Noninvasive techniques for glymphatic system modulation that hold promise for the treatment of ME/CFS include visual circuit activation (low-intensity 40 hertz blue light) [194], focused ultrasound combined with microbubbles [195], and multisensory gamma stimulation [196]; lymphatic–venous anastomosis is another consideration but is a much more invasive procedure [197]. Additional glymphatic modulation drugs that may also improve astrocyte and AQP4 function in ME/CFS include atipamezole, escitalopram [65], and systemic dexmedetomidine (promotes slow-wave activity, with a ∼32% increase in tracer influx and a ∼6-fold increase in hippocampal clearance) [198].

## 24. Concluding Remarks

In this article, we attempted to provide our perspective on the potential relationship between glymphatic dysfunction and the key symptoms of ME/CFS. The ideas presented here suggest commonalities in some of their essential features that may be useful to the ME/CFS scientific/medical community for deeper insight into the disease and better patient treatment.

ME/CFS has had a complex history, having been dismissed as a true pathology before being recognized as a biological disease. More recently, the ME/CFS scientific community has been interested in establishing the molecular basis of this disease, while being focused on several key areas, including the function of the immune system, metabolomics, muscle fatigue, and the nervous system. Even now, after all the efforts made during the past decades, an estimated 90% of people with ME/CFS are believed to be undiagnosed due to an absence of simple diagnostic tests [60]. Symptom overlap and comorbidities highlight the complexity of the disease, which suggest that a better understanding of its pathophysiology is urgently needed.

ME/CFS is now generally considered a neuroimmune disorder, involving interaction of neurological and immunological processes [199], and an overactive innate immune response [12]. Moreover, there is evidence of immune exhaustion [200], which may underlie many of the symptoms. ME/CFS is frequently characterized by PEM, where physical or mental exertion may lead to a worsening of symptoms, referred to as a “crash.” Other characteristic symptoms include unrefreshing sleep, fatigue, muscle pain, headache, orthostatic intolerance, and hypersensitivity to sensory stimuli. Of these, PEM and sleep disturbances are the hallmark symptoms of ME/CFS. With the recent discovery of the glymphatic system providing a mechanism for the removal of metabolic waste products from the brain, primarily during sleep, we were interested in exploring the relationship between GD and symptoms of ME/CFS. The details presented above suggest that glymphatic dysfunction may indeed be connected to the main characteristics of ME/CFS and may play a pivotal role in contributing to its pathophysiology. Therefore, we hope our approach will provide a clearer picture of the neurological component of ME/CFS. Attempts towards developing therapeutics for GD have already been initiated, the results of which may be of value for the effective treatment of ME/CFS, with an increased chance of recovery from the “crash” and ultimately a cure for this debilitating, chronic, and complex illness.

## Data Availability

No new data were created or analyzed in this study. Data sharing is not applicable to this article.
